# Early changes in rpS6 phosphorylation and BH3 profiling predict response to chemotherapy in AML cells

**DOI:** 10.1371/journal.pone.0196805

**Published:** 2018-05-03

**Authors:** Martin Grundy, Thomas Jones, Liban Elmi, Michael Hall, Adam Graham, Nigel Russell, Monica Pallis

**Affiliations:** 1 Clinical Haematology, Nottingham University Hospitals, Nottingham, United Kingdom; 2 Department of Haematology, Division of Cancer and Stem Cells, University of Nottingham, Nottingham, United Kingdom; Queen's University Belfast, UNITED KINGDOM

## Abstract

Blasts from different patients with acute myeloid leukemia (AML) vary in the agent(s) to which they are most responsive. With a myriad of novel agents to evaluate, there is a lack of predictive biomarkers to precisely assign targeted therapies to individual patients. Primary AML cells often survive poorly *in vitro*, thus confounding conventional cytotoxicity assays. The purpose of this work was to assess the potential of two same-day functional predictive assays in AML cell lines to predict long-term response to chemotherapy. (i) Ribosomal protein S6 (rpS6) is a downstream substrate of PI3K/akt/mTOR/ kinase and MAPK kinase pathways and its dephosphorylation is also triggered by DNA double strand breaks. Phospho-rpS6 is reliably measurable by flow cytometry and thus has the potential to function as a biomarker of responsiveness to several therapeutic agents. (ii) A cell’s propensity for apoptosis can be interrogated via a functional assay termed “Dynamic BH3 Profiling” in which mitochondrial outer membrane permeabilization in drug-treated cells can be driven by pro-apoptotic BH3 domain peptides such as PUMA-BH3. The extent to which a particular cell is primed for apoptosis by the drug can be determined by measuring the amount of cytochrome C released on addition of BH3 peptide. We demonstrate that phospho-rpS6 expression and PUMA-BH3 peptide-induced cytochrome C release after 4 hours both predict long term chemoresponsiveness to tyrosine kinase inhibitors and DNA double strand break inducers in AML cell lines. We also describe changes in expression levels of the prosurvival BCL-2 family member Mcl-1 and the pro-apoptotic protein BIM after short term drug culture.

## Introduction

AML is a heterogeneous clonal disorder of haemopoietic progenitor cells where both failure to differentiate and over proliferation results in accumulation of non-functional cells termed myeloblasts.[1] While nearly 80% of younger AML patients may initially achieve complete remission with current therapy, most will relapse with resistant disease.[2] Clinical outcomes in the elderly are even more modest as these patients do not tend to tolerate intensive chemotherapy regimens and frequently have adverse cytogenetics.[1]

There are many obstacles a chemotherapeutic drug has to circumvent before it can kill a leukaemia cell. Briefly, the drug has to reach its target, initiate the appropriate pro-apoptotic signals and overcome the cell’s anti-apoptotic defences. Despite the abundance of novel agents that have the potential to improve patient outcome, we are still lacking assays that clinicians can be offered to demonstrate which drugs the individual patient will best respond to. Most efforts in assigning therapy involve predicting a patient’s response to an agent based on their cytogenetic profile. The obvious solution might be chemosensitivity assays, but these have been tried, tested and found wanting.[3, 4] A confounding issue is that cells can be fragile *ex vivo*, and most AML cells will die spontaneously in culture fairly quickly (median survival at 48 hours = 38% of baseline).[5] Assays have been developed to maintain leukaemia cells *in vitro*, but AML samples are so heterogeneous that there is no “one-size-fits all” methodology for keeping them alive once they have been isolated from the patient. By focusing on same-day assays, with intact cells, we hope to overcome this obstacle.

Ribosomal protein S6 is a downstream substrate of PI3K/akt/mTOR/ p70S6 kinase and MAPK/p90S6 kinase pathways and is also dephosphorylated following DNA double strand breaks.[6–10] RpS6 is a constituent of the 40S ribosomal subunit that is phosphorylated at several sites including serines 235/236 and 240/244 upon activation by p70S6 and p90S6 kinases.[6] Phosphorylation of rpS6 controls mRNA translation in dividing cells,[11] and its recruitment to the 7-methylguanosine cap structure suggests a role in regulating assembly of the translation preinitiation complex.[8] The AKT and/or MAPK signalling pathways are constitutively active in the majority of AML cases.[12, 13] We have recently determined that rpS6 is hyperphosphorylated in AML patient samples, with phosphorylation being over 20 fold higher than in normal mobilised CD34+ cells.[14] Moreover, rpS6 phosphosrylation in patient samples can be abrogated by AKT and/or ERK inhibitors.[15] Some types of AML therapy, such as DNA damaging agents and receptor kinase inhibitors, might be expected to converge to dephosphorylate rpS6 through their actions on the akt/mTOR, ATM/AMPK/mTOR and/or ERK pathways.[9, 16] Phosphorylated rpS6 is therefore a potential biomarker of responsiveness to several therapeutic agents. Antibodies to rpS6 phosphorylated at serine 235/236 have been optimised for flow cytometry, where they are well-established as biomarkers for mTORC1 activity.[17, 18]

The B-Cell Lymphoma-2 (Bcl-2) family of proteins act at the mitochondria and regulate the internal apoptotic pathway. For apoptosis to occur, the proapoptotic effector molecules BAK and BAX must oligomerise and form pores that cause mitochondrial outer membrane permeabilisation (MOMP) resulting in cytochrome c release. Effector molecule activation can be triggered by BH3-only proapoptotic BCL-2 family members such as PUMA, BIM and BID.[19] These pro-apoptotic family members are usually sequestered by BCL-2 family prosurvival members such as MCL-1, BCL-2, and BCL-X_L_ which serves to hold apoptosis in check.[20] A cell’s propensity for apoptosis can be interrogated via a functional assay termed “Dynamic BH3 Profiling”.[21] Dynamic BH3 profiling can predict cellular responses to therapy based on measuring the capacity of drugs to prime mitochondria for apoptosis. The technique involves the addition of permeable pro-apoptotic BH3 peptides to drug primed cells to induce rapid mitochondrial outer membrane permeabilisation. The extent to which a particular cell is primed for apoptosis can be determined by measuring the amount of cytochrome c released. [22] Dynamic BH3 profiling using imatinib (with a 16 hour incubation) was found to predict clinical responsiveness in CML patients.[21]

In this study we assess whether short term (4 hour) rpS6 de-phosphorylation and/or PUMA-BH3 peptide-driven cytochrome c release can predict long-term (48 hour) response to chemotherapeutic drugs. As primary AML cells are unstable *in vitro* we utilize a panel of AML cell lines in order to obtain robust 48 hour IC_50_ values for reliable comparison with the short term functional assays. We also investigate whether drug exposure induces rapid changes in expression levels of Bcl-2 protein family members.

## Materials and methods

### Materials

Drugs and suppliers used in the study were as follows: 17-AAG, rapamycin, sorafenib, U0126 and torin 1 from LC labs (www.lclabs.com); AC220 and vosaroxin from Selleck (supplied by Stratech UK); etoposide from Tocris; gemtuzumab ozogamicin (GO) was a gift from Wyeth, Pearl River USA. C2 ceramide and Calyculin A were from Santa Cruz Biotechnology, Santa Cruz, CA, USA. Ly294006 was from Millipore, Watford, UK. Other drugs and reagents were from Sigma (Poole, Dorset, UK) unless specified.

### Cells

OCI-AML3, MOLM-13 and M-07e myeloid leukaemia cell lines were obtained from the German Collection of Microorganisms and Cell Cultures (DSMZ, Braunschweig, Germany). U937 and KG1a cell lines were from the European Collection of Animal Cell Cultures (Salisbury, UK). MV4-11 and TF-1a cells were obtained from the American Type Culture Collection (Manassas, VA, USA). HL-60 cells were a gift from Dawn Bradbury (Nottingham University Hospitals, UK), OCI-AML6.2 cells were a gift from Dr. Jo Mountford (University of Glasgow, UK), M0-91 cells were a gift from Joseph Scandura (Cornell Medical College, USA). OCI-AMLDNR cells were developed in our laboratory.[23] HL-60, U937, OCI-AML3, OCI-AMLDNR, OCI-AML6.2, MOLM-13, TF-1a, M0-91 and MV4-11 cell lines were maintained in RPMI 1640 medium with 10% foetal calf serum (FCS; First Link, Birmingham, UK), 2mM L-glutamine, 100 U/ml penicillin and 10μg/ml streptomycin. The KG1a and M-07e cell lines were maintained as above with 20% FCS and the M-07e having the addition of 10ng/ml GM-CSF (Novartis, Basel, Switzerland). All cultures were kept at 37°C in 5% CO_2_ and all experiments were performed with cell lines in log phase. Regular testing to authenticate these cell lines was performed using multiplex short tandem repeat analysis (Powerplex 16; Promega, Southampton, UK). Mycoplasma testing was carried out routinely using the Mycoalert mycoplasma detection kit (Lonza, Rockland, USA) and following the manufacturer’s instructions.

### Chemosensitivity assay

Cells were plated in triplicate at 2.5x10^5^/ml with drug or untreated controls in 96 well plates. Plates were incubated for 48 hours at 37°C in 5% CO2 with the addition of alamar blue (Serotec, BUF012A) for the final 4 hours. Fluorescence was recorded using a POLARstar optima plate reader (BMG technologies, UK). Cell lines were deemed sensitive or resistant to each agent using the following criteria (<5 X 10^th^ centile IC_50_ = sensitive; >5 X 10^th^ centile IC_50_ = resistant).

### Phospho-S6 ribosomal protein expression

Cells were incubated at 5x10^5^/ml in culture medium for four hours with the indicated drugs. Phospho-S6 ribosomal protein expression (using Alexa-647-conjugated rpS6 p-ser235/236 antibody, CST #4851) was measured following fixation in 2% paraformaldehyde and permeabilisation with 0.1% saponin as described.[14] Baseline rpS6 phosphorylation was determined by culturing with the mTOR inhibitors rapamycin (100 nM) and torin1 (1 μM) and the ERK inhibitor U0126 (3 μM). Adjustments for baseline rpS6 phosphorylation and expression in untreated cells were made using the calculation %rpS6 dephosphorylation = 100 - 100X (MFI with agent–baseline MFI)/(untreated MFI–baseline MFI), where MFI = mean fluorescence intensity.

### Dynamic BH3 profiling

Cells were incubated at 5x10^5^/ml in culture medium for four hours with the indicated drugs. Cytochrome c release (using Alexa-647-conjugated cytochrome c antibody, Becton Dickinson #558709) was measured after a further 60 minute incubation of digitonin-permeabilised cells with PUMA-BH3 peptide as described.[22, 24] In preliminary assays, the PUMA-BH3 was optimised to 3 μM in all cells except M-091, as this was the concentration of the peptide found to be sufficient to induce mitochondrial outer membrane permeabilisation in drug-primed cells, but not so high that it induced a high degree of mitochondrial outer membrane permeabilisation without drugs. In M-091, the peptide was used at 0.5 μM. Adjustments for peptide-induced cytochrome c release in untreated cells were made in order to establish agent-specific release, using the formula 100X (percent cytochrome c positive with peptide–percent cytochrome c positive with drug plus peptide)/(percent cytochrome c positive with peptide). A mutated PUMA-BH3 peptide (PUMA2A) [22] at 100 μM and BIM-BH3 peptide (10 μM) were used as controls in all experiments. Data were collected on a FACSCanto II flow cytometer (Becton Dickinson) and analysed with FACS Diva software (Becton Dickinson).

### Western blot analysis

MV4-11 cells were treated for four hours with 1 μM etoposide, 10 nM AC220 or 1 μM torin1. Cell lysates were prepared, separated by sodium dodecyl sulfate polyacrylamide gel electrophoresis, and transferred to nitrocellulose membranes. Detection antibodies included anti-β-Actin (N-21, sc-130656), anti-MCL1 (S-19, sc-819) and anti-Bcl-2 (C-2, sc-7382) from Santa Cruz Biotechnology, Santa Cruz, CA, USA, anti-BID (#2002, Cell Signalling Technology, Leiden, Netherlands), anti-BIM (Y36, ab32158), anti-BAD (phospho S136, ab28824) and anti-PUMA (EP512Y, ab33906) from Abcam, Cambridge, UK.

### Calculations and statistics

Statistical analysis was carried out using the Statistical Package for Social Sciences, version 23 (SPSS, Chicago, IL, USA). P values of ≤0.05 were considered to represent significance. Area under curve (AUC) receiver-operating characteristic (ROC) predictive analysis was used to evaluate the relationship between sensitivity of cell lines to an agent and rpS6 phosphorylation or cytochrome c release.[25]

## Results

### Multiple signalling pathways converge on rpS6 phosphorylation

**Fig 1:**
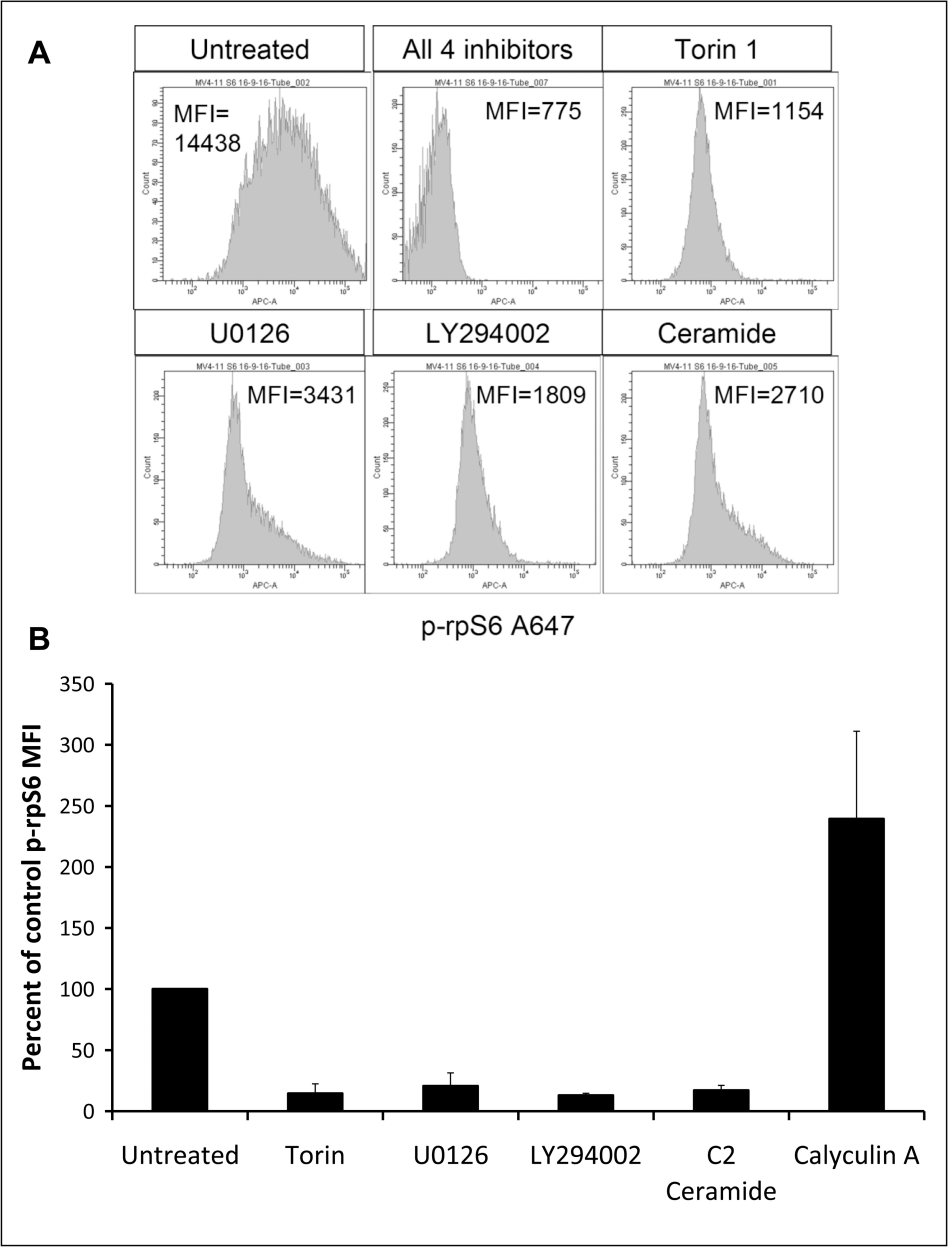
RpS6 dephosphorylation by signalling inhibitors. **(A)** MV4-11 cells were cultured for 4 hours with 1 μM torin1, 5 μM U0126, 30 μM LY294002, 30 μM C2-ceramide or a combination of all 4 inhibitors. **(B)** Values are a percent of untreated cell phospo-rpS6 as described in the methods. (Mean+/- SD for n = 3–5). The phosphatase inhibitor Calyculin A (3 nM) was used as positive control.

The MAPK and/or AKT signalling pathways are constitutively active in the majority of AML cases.[12, 13] RpS6 is a downstream mediator of both pathways,[7, 8] and is constitutively phosphorylated in patient samples.[14] P-rpS6 has been used previously as a sensitive marker for mTORC1 activation in flow cytometric studies.[15, 17, 18] To examine basal phosphorylation and inhibitor-induced dephosphoryation of rpS6 in our assay system, we cultured MV4-11s for 4 hours with the mTORC1 inhibitor torin1, the ERK inhibitor U0126 and the akt inhibitor LY294002. All these agents were able to greatly reduce rpS6 phosphorylation (Fig 1). The cytotoxic drug-activated signalling lipid ceramide [26–28] was also inhibitory. In contrast, the phosphatase inhibitor calyculin A enhanced S6 phosphorylation.

### RpS6 dephosphorylation at 4 hours predicts 48 hour drug sensitivity

Data from Fig 1 suggests early changes in rpS6 phosphorylation may be used as a predictor of chemotherapeutic response in AML cells. To test this hypothesis, 11 AML cell lines were subjected to 48 hour dose response assays with the following agents: FLT3 inhibitors (AC220 and sorafenib),[29, 30] DNA double strand break (DSB) inducers (etoposide, GO and vosaroxin),[31–33] the standard-of-care drug cytarabine and the hsp90 inhibitor 17-AAG.[34]

**Fig 2:**
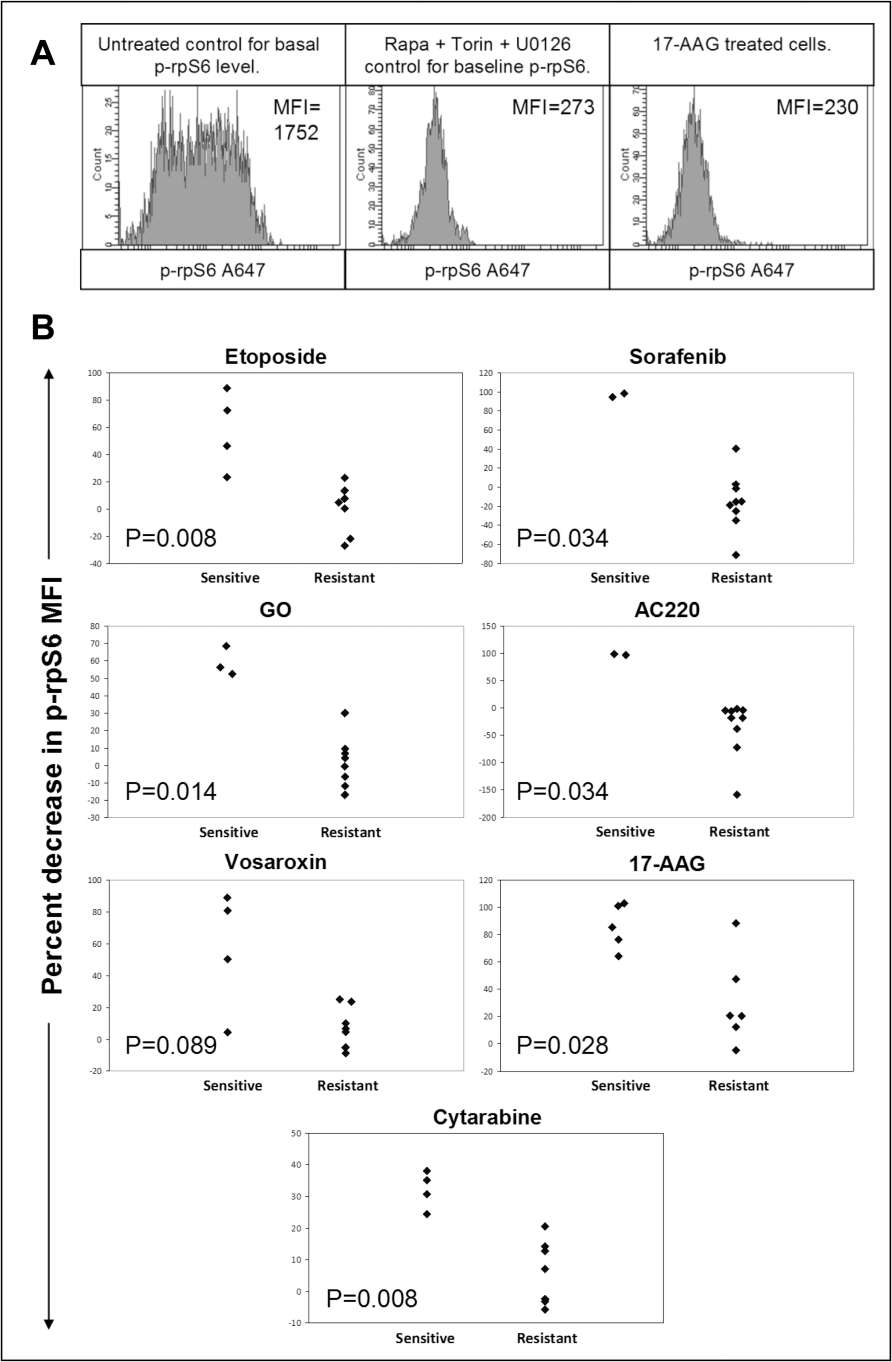
RpS6 dephosphorylation at 4 hours predicts 48 hour drug sensitivity. **(A)** MOLM-13 cells were cultured for 4 hours with 100 nM Rapamycin, 1 μM torin1 and 3 μM U0126 to determine p-rpS6 baseline. RpS6 dephosphorylation after 4 hours culture with 500 nM 17-AAG is also shown. Example histograms are representative of 3 individual experiments. **(B)** Based on the 48 hour IC_50_ values a drug sensitive/resistant cut off for cell lines was determined. Cell lines were cultured for 4 hours with 1 μM etoposide, 50nM sorafenib, 600ng/ml GO, 10nM AC220, 1 μM vosaroxin, 500nM 17-AAG and 2 μM cytarabine. Values are a percent of untreated cell p-rpS6 as described in the methods. Each point represents a cell line and is the product of three individual experiments.

Using ROC analysis (predictive accuracy test), rpS6 dephosphorylation at 4 hours predicted the 48 hour response to the DSB inducing drugs etoposide and GO, the FLT3 inhibitors sorafenib and ACC220 as well as to cytarabine (Fig 2 and S1 Table). ROC analysis of 4 hour rpS6 dephosphorylation compared to 48 hour drug sensitivity confirmed that the assay was highly sensitive and specific with AUC values of 1.0 for etoposide, GO, sorafenib, AC220 and cytarabine (S1 Fig shows individual ROC curves). An AUC value close to 1 indicates excellent predictive capabilities providing evidence that this laboratory test performed after 4 hours does have an ability to predict chemosensitivity at 48 hours. For vosaroxin, the AUC value was 0.82. HL-60 cells, which were sensitive to vosaroxin at 48 hours, did not dephosphorylate rpS6.

17-AAG, which had been included because of clinical interest in novel hsp90 inhibitors, [35] was expected to be particularly effective because hsp90 plays a direct role in maintatining the stability of rpS6.[36] The predictive value for 17-AAG (AUC 0.9) was statistically significant, albeit with U937 cells showing confoundingly high dephosphorylation.

### PUMA-BH3 peptide-induced cytochrome c release after 4 hours drug treatment predicts 48 hour drug sensitivity

**Fig 3:**
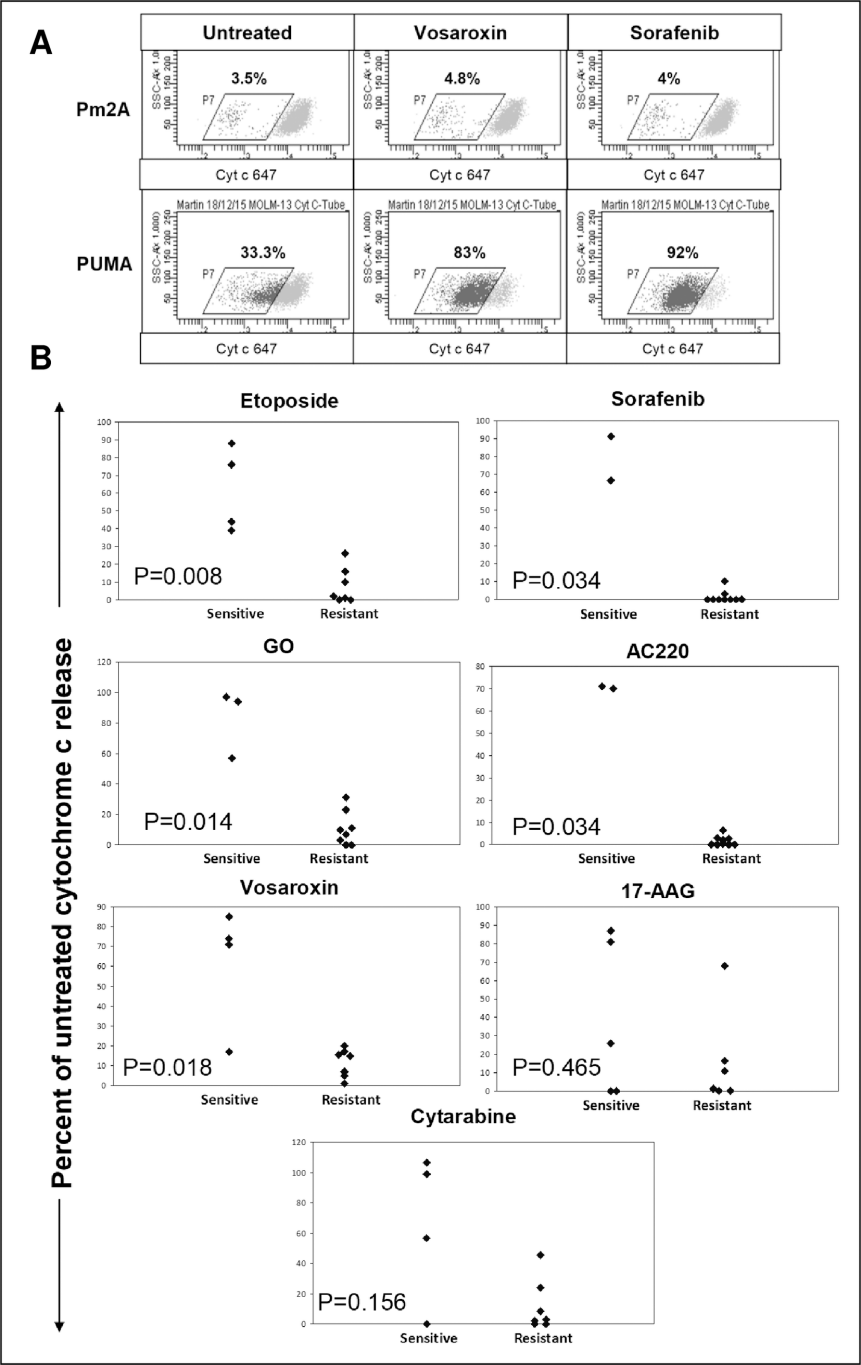
PUMA-BH3 peptide-induced cytochrome c release after 4 hours drug treatment predicts 48 hour drug sensitivity. **(A)** MOLM-13 cells were cultured for 4 hours with 1 μM vosaroxin or 50 nM sorafenib followed by 1 hour treatment with PUMA-BH3 peptide or PUMA2A control. Example dot plots are representative of 3 individual experiments. **(B)** Based on the 48 hour IC_50_ values a drug sensitive/resistant cut off for cell lines was determined as described in the methods. Cell lines were cultured for 4 hours with 1 μM etoposide, 50nM sorafenib, 600ng/ml GO, 10nM AC220, 1 μM vosaroxin, 500nM 17-AAG or 2 μM cytarabine followed by 1 hour incubation with PUMA-BH3 peptide. Values are corrected for cytochrome c release with PUMA2A control peptide as described in the methods. Each point represents a cell line and is the product of three individual experiments.

Dynamic BH3 profiling involves exposing mitochondria to BH3 domain derived peptides following short term drug exposure to prime mitochondria for changes in MOMP as measured by cytochrome c release. It has been demonstrated that this technique can predict cytotoxicity.[21] PUMA-BH3 peptide can sensitise all the anti-apoptotic BCL-2 family of proteins [37] and was used for our assay. To obtain optimal sensitivity with this technique it is important to establish suitable drug and peptide concentrations so that agent or peptides do not overwhelm the system by inducing too much cytochrome c release individually. As the technique demands outer membrane permeabilisation whilst maintaining mitochondrial integrity, we set up appropriate assay controls and defined their interpretation as >90% induced cytochrome c release in the presence of a high concentration (10 μM) of the activator peptide BIM-BH3 but less than 10% when incubated with the non-specific peptide PUMA2A-BH3. PUMA-BH3 peptide-induced cytochrome c release closely predicted the 48 hour IC_50_ for DSB inducing drugs and FLT3 inhibitors, with AUC values of 1.0 for etoposide, GO, AC220 and sorafenib (Fig 3 and S1 Table). For vosaroxin, the AUC value was 0.95. As with the rpS6 assay, HL-60 cells, which were sensitive to vosaroxin at 48 hours, were insensitive in the short-term assay. BH3 profiling was not predictive for responses to 17-AAG, (AUC value 0.63), or cytarabine (AUC value 0.77). See S2 Fig for individual ROC curves.

**Fig 4:**
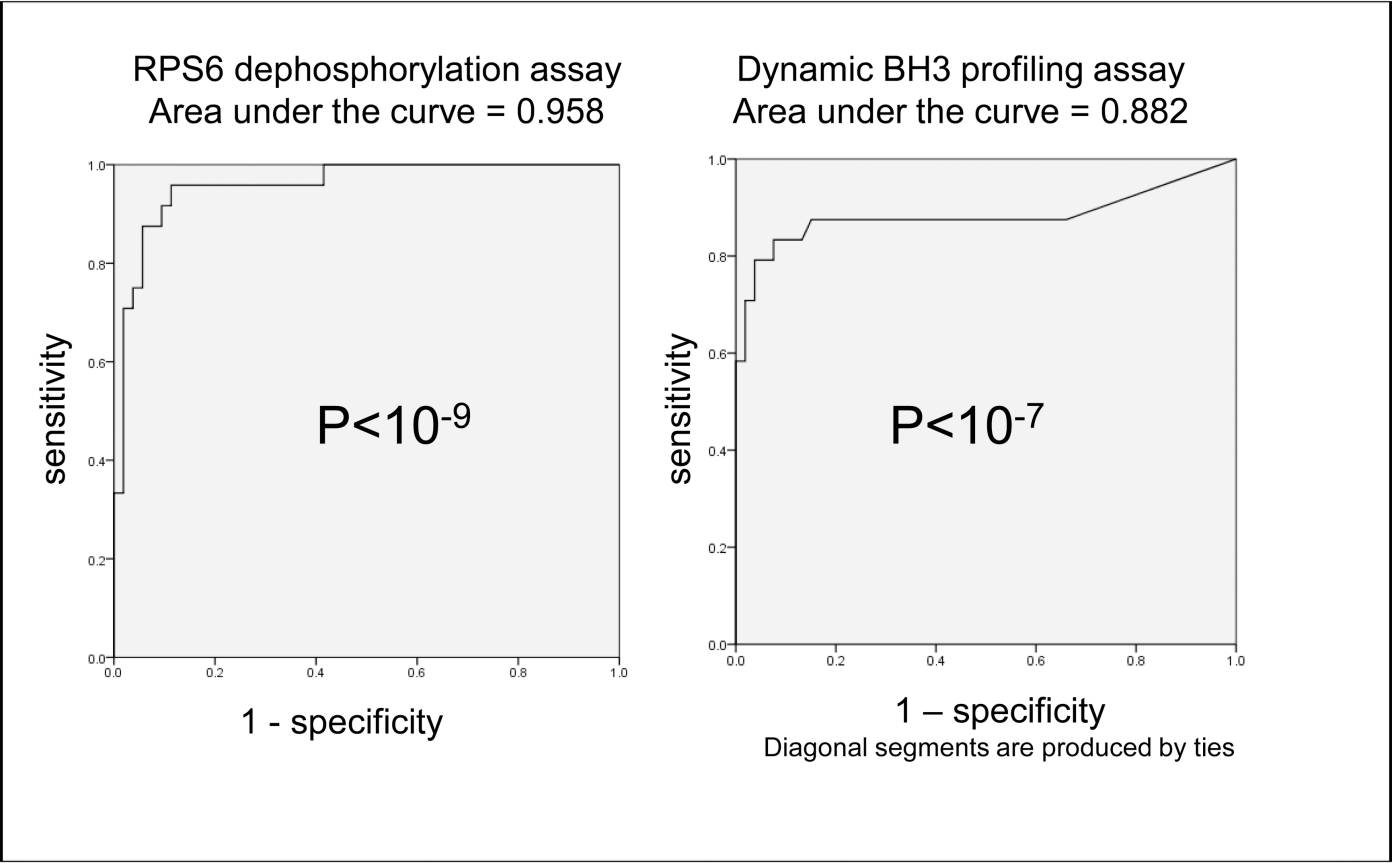
ROC curve analysis confirms highly significant overall sensitivity and specificity for the ability of both rpS6 dephosphorylation and PUMA-BH3 induced cytochrome C release after 4 hours drug treatment to predict 48 hour sensitivity to drugs. Summary ROC curves for percent change in rpS6 phosphorylation and PUMA induced cytochrome c release after 4 hours treatment with 1 μM etoposide, 50nM sorafenib, 600ng/ml GO, 10nM AC220, 1 μM vosaroxin, 500nM 17-AAG or 2 μM cytarabine in 11 AML cells lines. Cell lines were classified as sensitive or resistant according to 48 hours drug response (The standardised definition of sensitivity is described in the methods section). Each data point used to generate the analysis is the mean of three individual experiments.

Summarising the ROC analysis across the 11 cell lines and 7 drugs (Fig 4), both rpS6 dephosphorylation and dynamic BH3 profiling assays showed highly significant predictive ability.

### Changes in expression of apoptotic modulator proteins after four hours drug exposure

**Fig 5:**
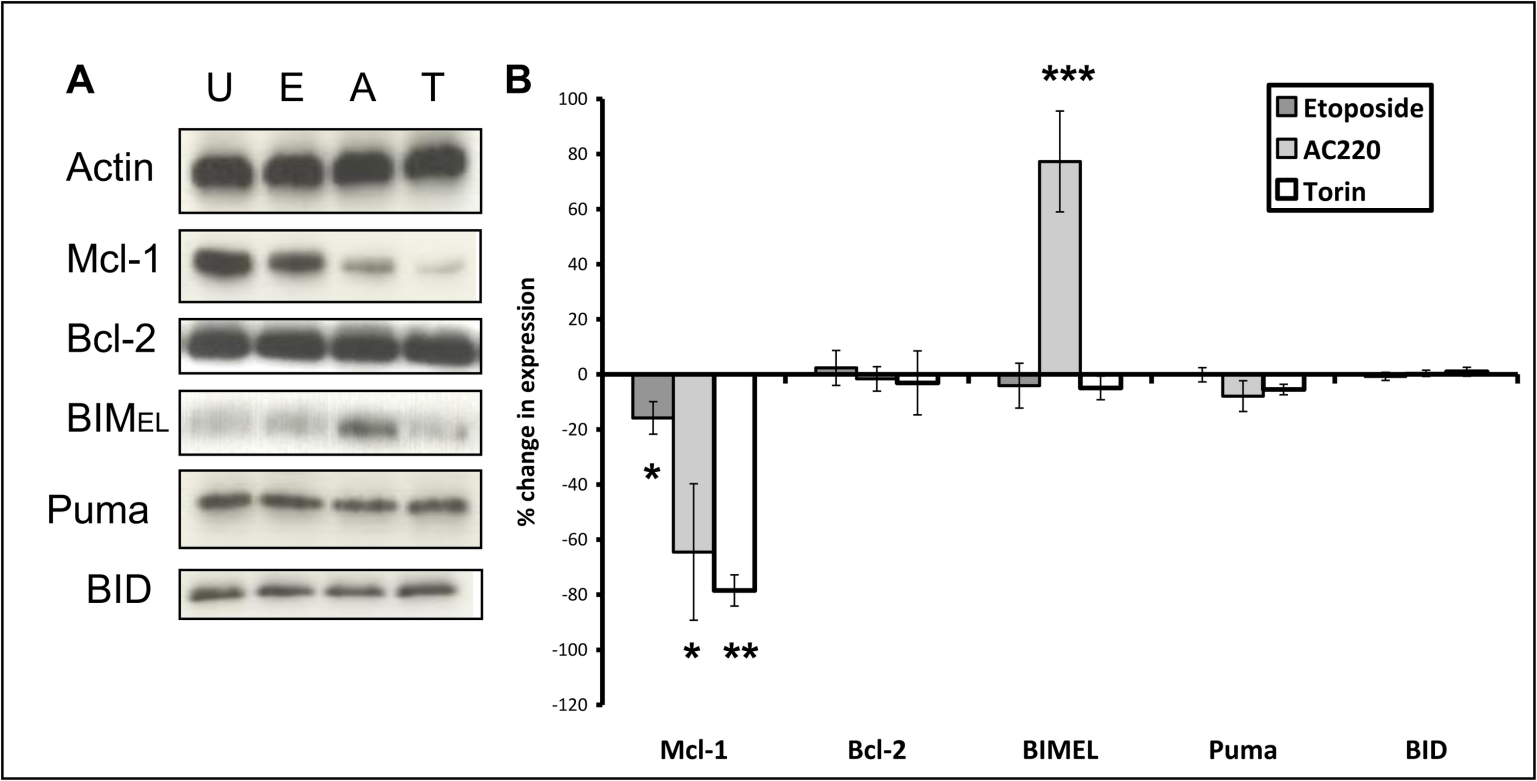
Changes in expression of apoptotic modulator proteins after four hours drug exposure. **(A)** MV4-11 cells were treated for four hours with 1 μM etoposide (E), 10 nM AC220 (A) or 1 μM torin1 (T). Each blot represents one of three independent experiments. **(B)** Blots were subjected to densitometry analysis using Image Studio Lite software (version 5.2). Values shown are percent change in expression normalised to loading control (*p = <0.05/**p = 0.002/***p = 0.02), analyzed using paired samples t-test, +95% confidence interval. Columns, mean of three experiments; bars, SD.

For apoptosis to occur, the effector molecules BAK and BAX oligomerise and form pores that cause MOMP, resulting in cytochrome c release. Effector molecule activation can be triggered by the BH3-only proapoptotic BCL-2 family members BIM, BID and PUMA. Prosurvival members such as MCL-1, BCL-2 and BCL-X_L_ inhibit the BH3-only proteins by sequestration and hold apoptosis in check. Our dynamic BH3 profiling results clearly demonstrate that drugs are priming cells to PUMA-BH3 peptide after only 4 hours exposure. We investigated what effect the drugs were having on BCL-2 apoptotic protein family members during this time period that might underpin this rapid priming. MV4-11 cells were used as these cells were the most sensitive cell line to the majority of drugs in our panel (S1 Table). These cells do not over-express the anti-apoptotic protein BCL-X_L_.[24] Of the other BCL-2 family prosurvival members MCL-1 has the shorter half-life (approximately 1 hour) and can be rapidly downregulated [38] whilst BCL-2 is a much more stable protein.[37, 39] We used etoposide and AC220, as DSB-inducing agents and FLT3 inhibitors are of particular clinical interest in AML: both rpS6 dephosphorylation and dynamic BH3 profiling had been able to predict response to these drugs (S1 Table). We found that etoposide and AC220 significantly deplete MCL-1 in the MV4.11 cells without affecting the expression of BCL-2 (Fig 5). The mTORC1 antagonist and translation inhibitor torin1 [40] was included as a control because inhibition of translation depletes MCL-1.[41] Activation of effector molecules BAK and BAX using conformation-specific antibodies as previously described [24] was not observed (data not shown). Of the activator BCL-2 family members BID, BIM and PUMA, BIM expression was significantly increased after 4 hours treatment with AC220 only whilst BID and PUMA were unaffected by either etoposide or AC220 (Fig 5). See S3 Fig for uncropped blots.

## Discussion

This work is a first step towards ascertaining whether short term functional predictive assays might have clinical application in AML. A history of failure for chemosensitivity assays in clinical practice [3] has been discouraging, but the need for the development of such assays is still being strongly asserted,[4, 42, 43] with the rationale that molecular assays in unstimulated cells cannot recapitulate complex behaviour.

We have demonstrated the potential of two same-day functional flow cytometric assays to predict 48-hour response to chemotherapy in AML cell lines. FLT3 inhibitors are documented to induce rapid changes in signalling pathways [44–46] and we have shown here that rpS6 dephosphorylation is a suitable indicator of this activity. Early (i.e. less than four hours) pro-apoptotic changes invoked by DNA damaging drugs are rarely documented. However, in an exception to the general focus on late changes, Nijhawan and colleagues documented the early loss of MCL-1 after genotoxic stress, and they included etoposide in their analysis.[47] The rapidity of ceramide induction following DNA damage [26, 27] and the involvement of ceramide in phosphatase activation[48] also encouraged us to postulate that we might be able to document responses to DNA-damaging agents after four hours. The cell lines were appropriately classified as sensitive or resistant to etoposide and GO. Vosaroxin sensitivity was also predicted by both assays in 10/11 cell lines.

The main obstacle for further development of this work in clinical samples is that primary samples are unstable over time *in vitro* [5] and therefore cannot be used for proof of principle. Bolt-on studies in the context of clinical trials will be needed to properly evaluate the methodologies. Since AML samples can be hypocellular at presentation, the availability of cells can be a problem for diagnostic assays. Phospho-rpS6 can be performed on very small samples with minimal cell loss during processing. However, some primary samples have low basal rpS6 phosphorylation.[14] BH3 profiling requires permeabilisation of the outer cell membrane, while maintaining viable mitochondria: this is technically challenging [22] and the cell requirement includes positive and negative controls for permeabilisation as well as no peptide controls for each drug. BIM-BH3 has been used by others for profiling without drugs.[49, 50] Our choice of PUMA-BH3 rather than BIM-BH3 was made because PUMA gave a more consistent low baseline level of cytochrome release at a single concentration across several cell lines in preliminary experiments. In performing the assay in 11 cell lines for this study, we found that one (M0-91) was hypersentitive to PUMA-BH3 alone (see methods) and the whole assay had to be repeated with a lower peptide concentration. In summary, BH3 profiling requires more cells and may also require sample-specific optimisation of baseline conditions. The area under the curve in Fig 4 was lower for BH3 profiling than for rpS6 phsosphorylation. However, dynamic BH3 profiling is the more logical assay for early chemosensitivity testing as it takes into account apoptosis resistance that might occur separately from or downstream of pRS6 inhibition.

Perturbations of signalling pathways can induce drug resistance mechanisms,[51, 52] but the finding that rpS6 dephosphorylation at 4 hours was predictive of cell death at 48 hours implies early, irreversible changes. In the final part of the study we probed for early drug-induced changes in BCL-2 family proteins that might indicate commitment to apoptosis. Apoptosis is effected by oligomerisation of BAX and BAK and activated by BCL-2 family members such as BIM, BID and PUMA.[19] The only change we noted in these pro-apoptotic molecules was induction of BIM by AC220 in sensitive MV4-11 cells (Fig 5). We found significant early inhibition of MCL-1 by AC220 in these cells (Fig 5). This might be predicated on early downregulation of STAT5 signalling: [53] activation of this pathway is documented to maintain MCL-1 expression.[54] However, MCL-1 was only 20% decreased in etoposide-treated cells. Given that elimination of MCL1 is reported to be required for the initiation of apoptosis following double strand breaks[47] the delineation of early irreversible pre-apoptotic changes will likely require attention to post-translational changes and binding partners as well as expression levels of MCL-1. MCL-1 is sequentially translocated to mitochondria, phosphorylated and ubiquitinylated prior to degradation.[55] A possible explanation for the clear-cut predictive value of the assays in the face of resistance mechanisms is the homogeneity of cell lines. A future application of the system might be to elucidate whether sensitive and resistant subsets within patient samples can be defined.

In summary we have described two assays which detect changes occurring in sensitive cells within four hours of drug application and which predict sensitivity and resistance to DNA damaging agents and FLT3 inhibitors in a panel of AML cell lines.

## Supporting information

S1 Table Cell line IC50, rpS6 and cytochrome C data. 11 cell lines were treated with 7 different drugs or untreated controls for 48 hours to determine an IC50 (nM) (shown in bold; supscript R = resistant, superscript S = sensitive). Percent rpS6 dephosphorylation (regular font) and percent PUMA induced cytochrome C release (bold italic) was determined in the same cell lines after 4 hours drug treatment. Each value is the product of three individual experiments.
(TIF)

S1 Fig Individual ROC curves for rpS6 dephosphorylation after 4 hours drug treatment. ROC curves for percent change in rpS6 phosphorylation after 4 hours treatment with 1 μM etoposide, 50nM sorafenib, 600ng/ml GO, 10nM AC220, 1 μM vosaroxin, 500nM 17-AAG or 2 μM cytarabine in 11 AML cells lines. Each data point used to generate the analysis is the mean of three individual experiments.
(TIF)

S2 Fig Individual ROC curves for PUMA induced cytochrome c release after 4 hours drug treatment. ROC curves for PUMA induced cytochrome c release after 4 hours treatment with 1 μM etoposide, 50nM sorafenib, 600ng/ml GO, 10nM AC220, 1 μM vosaroxin, 500nM 17-AAG or 2 μM cytarabine in 11 AML cells lines. Each data point used to generate the analysis is the mean of three individual experiments.
(TIF)

S3 Fig Original uncropped western blots. MV4-11 cells were treated for four hours with 1 μM etoposide, 10 nM AC220 or 1 μM torin1 before probing for the apoptotic modulator proteins Mcl-1, Bcl-2, BIM, PUMA and BID.
(TIF)
