# Supplementary figures and images for: Early changes in rpS6 phosphorylation and BH3 profiling predict response to chemotherapy in AML cells
Source: PLoS One. 2018 May 3;13(5):e0196805. doi: 10.1371/journal.pone.0196805 (PMC5933738; doi:10.1371/journal.pone.0196805)

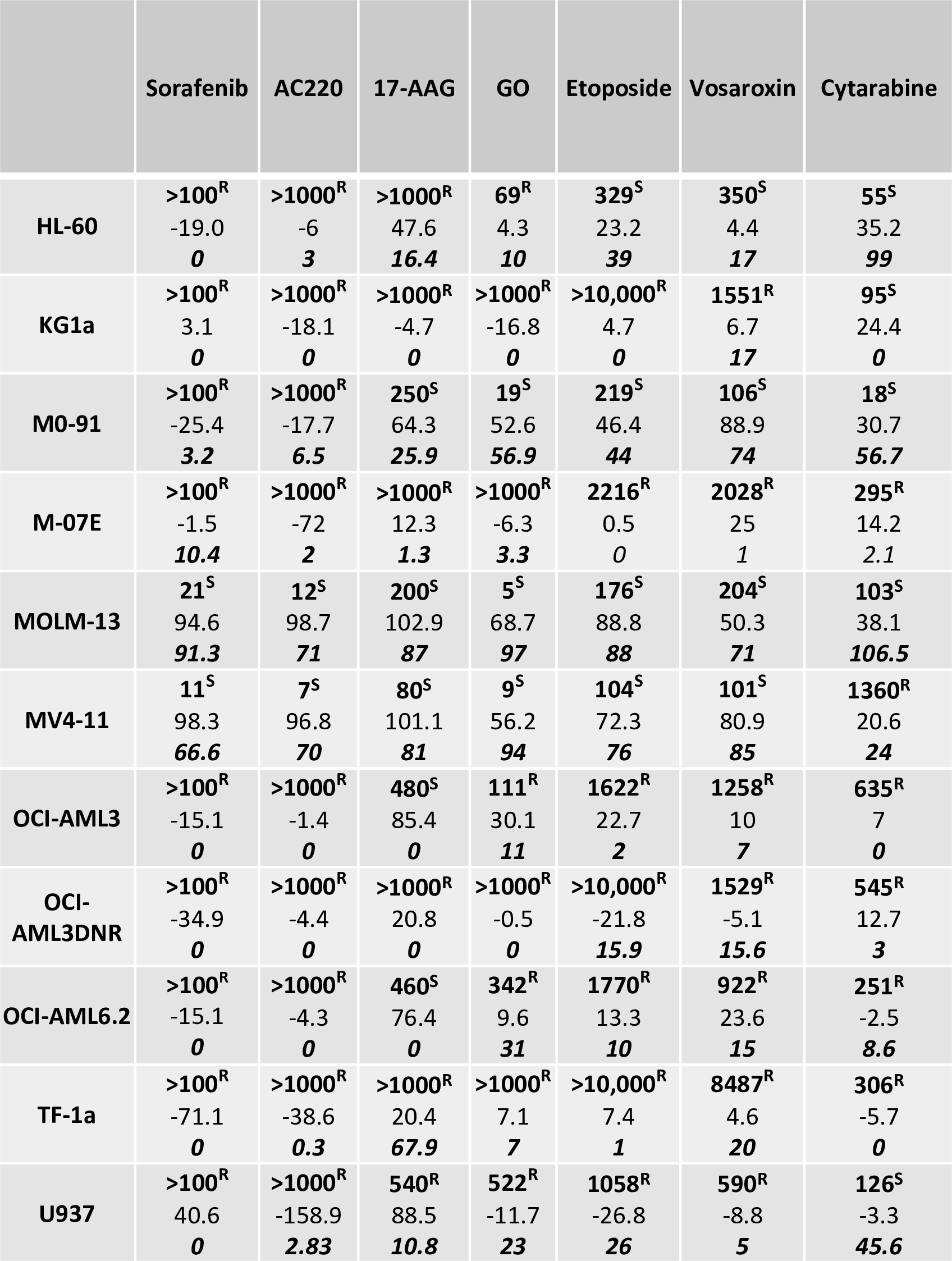

Supplement: S1 Table — 11 cell lines were treated with 7 different drugs or untreated controls for 48 hours to determine an IC50 (nM) (shown in bold; supscript R = resistant, superscript S = sensitive). Percent rpS6 dephosphorylation (regular font) and percent PUMA induced cytochrome C release (bold italic) was determined in the same cell lines after 4 hours drug treatment. Each value is the product of three individual experiments. (TIF) [file pone.0196805.s001.tif]

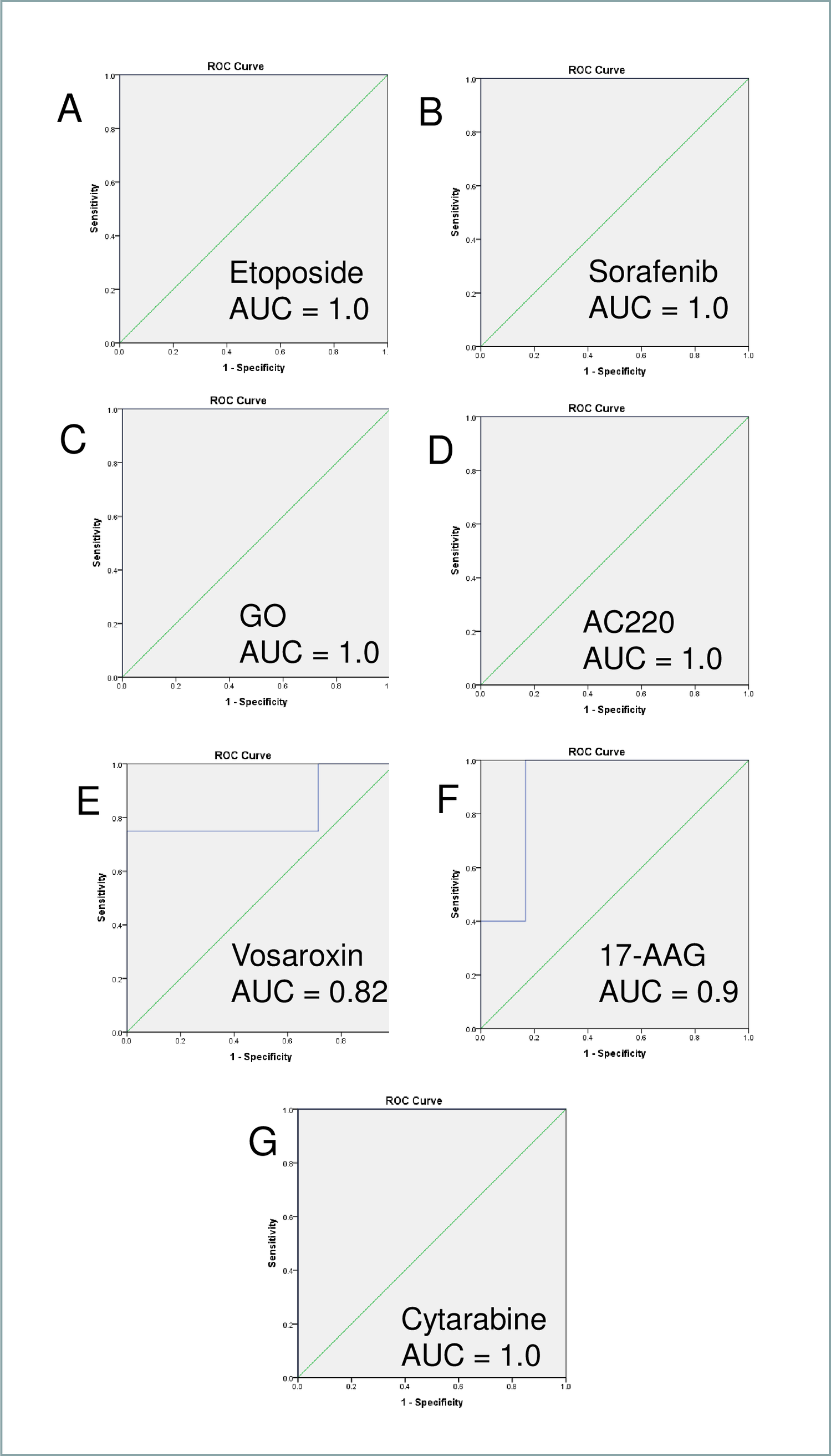

Supplement: S1 Fig — ROC curves for percent change in rpS6 phosphorylation after 4 hours treatment with 1 μM etoposide, 50nM sorafenib, 600ng/ml GO, 10nM AC220, 1 μM vosaroxin, 500nM 17-AAG or 2 μM cytarabine in 11 AML cells lines. Each data point used to generate the analysis is the mean of three individual experiments. (TIF) [file pone.0196805.s002.tif]

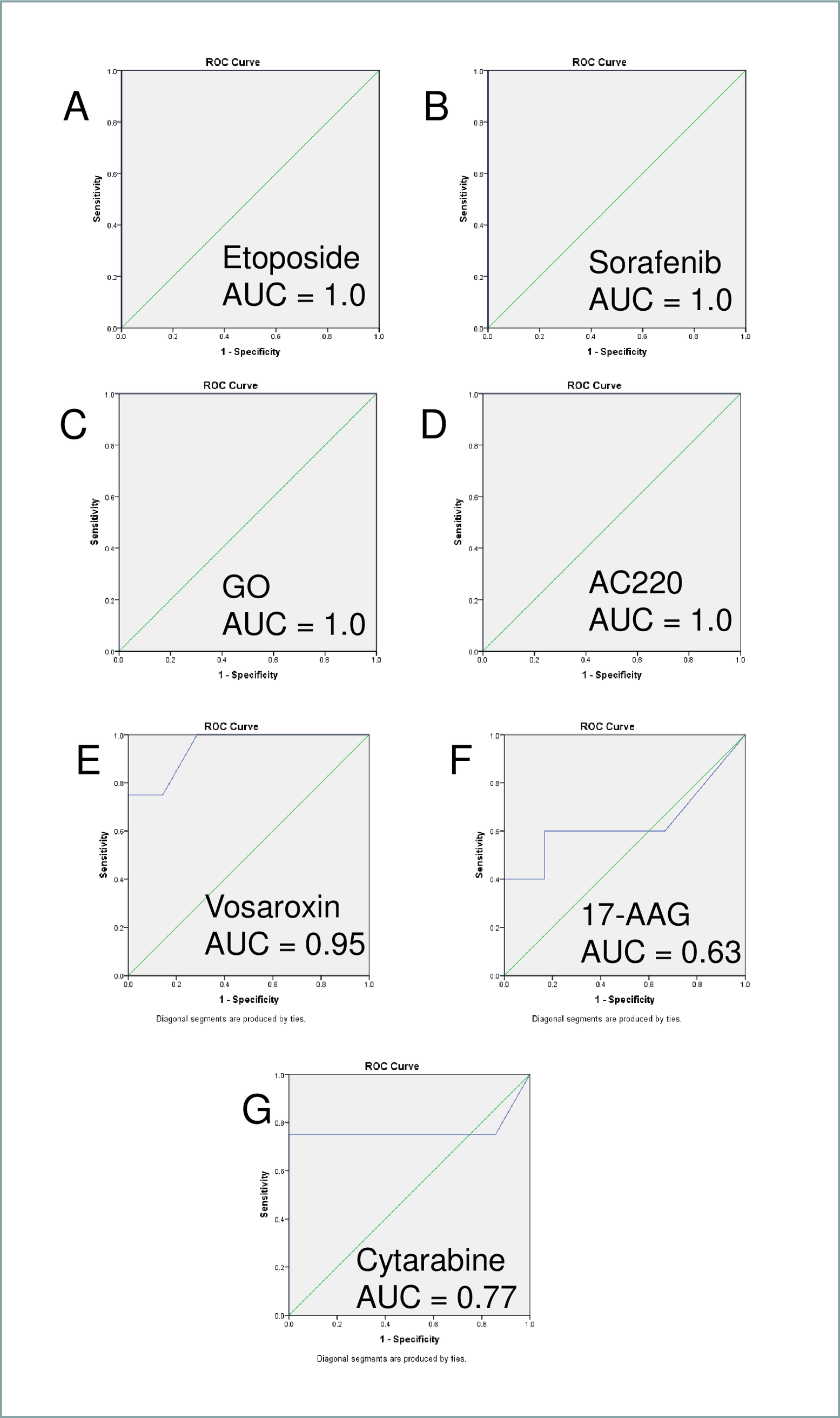

Supplement: S2 Fig — ROC curves for PUMA induced cytochrome c release after 4 hours treatment with 1 μM etoposide, 50nM sorafenib, 600ng/ml GO, 10nM AC220, 1 μM vosaroxin, 500nM 17-AAG or 2 μM cytarabine in 11 AML cells lines. Each data point used to generate the analysis is the mean of three individual experiments. (TIF) [file pone.0196805.s003.tif]

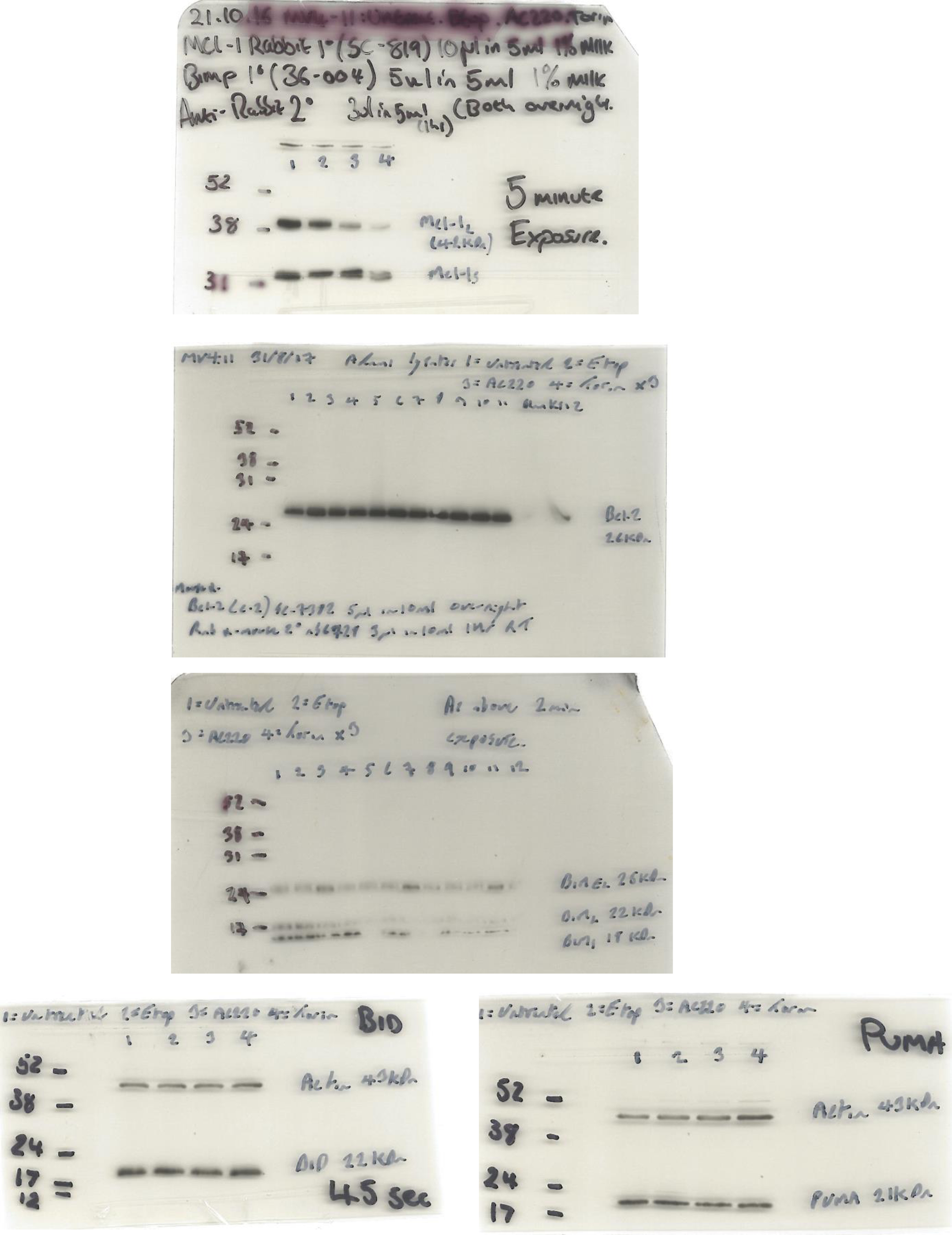

Supplement: S3 Fig — MV4-11 cells were treated for four hours with 1 μM etoposide, 10 nM AC220 or 1 μM torin1 before probing for the apoptotic modulator proteins Mcl-1, Bcl-2, BIM, PUMA and BID. (TIF) [file pone.0196805.s004.tif]
